# High Mobility Group Box Protein-1 in Wound Repair

**DOI:** 10.3390/cells1040699

**Published:** 2012-09-28

**Authors:** Elia Ranzato, Simona Martinotti, Marco Pedrazzi, Mauro Patrone

**Affiliations:** 1 Department of Sciences and Innovative Technology, (DiSIT), University of Piemonte Orientale "A. Avogadro", Viale Teresa Michel 11, Alessandria 15121, Italy; Email: simona.martinotti@unipmn.it (S.M.); mauro.patrone@unipmn.it (M.P.); 2 Molecular Histology and Cell Growth Laboratory, San Raffaele Science Institute, Via Olgettina 58, Milan 20132, Italy; 3 Department of Experimental Medicine (DIMES)-Biochemistry Section, Center of Excellence for Biomedical Research (CEBR), University of Genoa, Viale Benedetto XV, Genoa 1-16132, Italy; Email: marco.pedrazzi@unige.it

**Keywords:** HMGB1, alarmin, DAMP, tissue repair, wound cytokine

## Abstract

High-mobility group box 1 protein (HMGB1), a member of highly conserved non-histone DNA binding protein family, has been studied as transcription factor and growth factor. Secreted extracellularly by activated monocytes and macrophages or passively released by necrotic or damaged cells, extracellular HMGB1 is a potent mediator of inflammation. Extracellular HMGB1 has apparently contrasting biological actions: it sustains inflammation (with the possible establishment of autoimmunity or of self-maintaining tissue damage), but it also activates and recruits stem cells, boosting tissue repair. Here, we focus on the role of HMGB1 in physiological and pathological responses, the mechanisms by which it contributes to tissue repair and therapeutic strategies base on targeting HMGB1.

## 1. Introduction

This Review article focuses on the biology of high-mobility group box 1 protein (HMGB1; also known as amphoterin or HMG1), an evolutionarily ancient protein that was discovered recently to have also a role as a cytokine. HMGB1 probably originated more than 500 million years ago, before the split between the animal and plant kingdoms. The characteristic double HMG-box organization of HMGB1 that is found in metazoans indicates that HMGB1 probably had a cytokine-like role before extensive gene duplication occurred in the genome, which then resulted in the emergence of paired cytokines and cytokine receptors.

**Figure 1:**
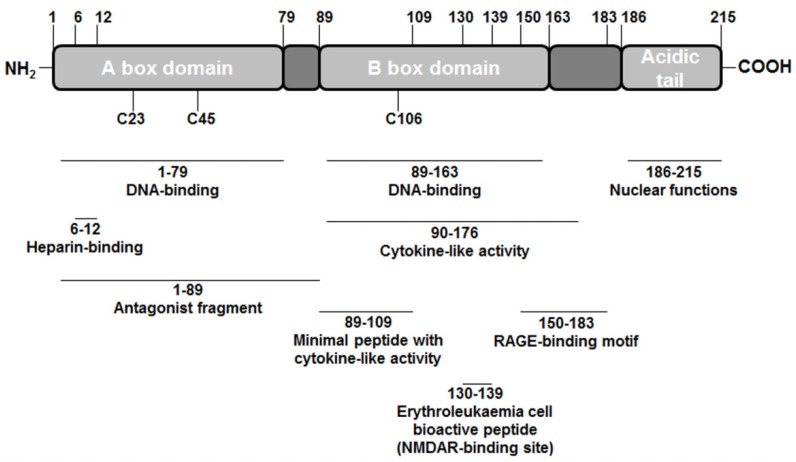
Scheme of high-mobility group box 1 (HMGB1) structure.

It was first identified in 1973 as one of a group of chromatin-associated protein with high acidic and basic amino acid content [1]. HMGB1 is composed of two tandem DNA binding HMG-box domains (N-terminal A and central B), and an acidic C-terminal tail, mediating protein-protein interactions (see Figure 1). This structure confers HMGB1 the peculiar feature to recognize and specifically bind DNA structures, containing sharp bends or kinks, such as four way junctions [2,3], DNA damaged by the anticancer drug cisplatin [4] and by UV-light [5], or to induce bending in linear duplex DNA. The structure-specific DNA binding is attributed to the A box [6] while the DNA bending capacity is primarily related to the B domain [7]. The abilities of HMGB1 to bend DNA and to bind bent DNA define this protein as an architectural factor, promoting the assembly of nucleoprotein complexes. This crucial role explains the implication of HMGB1 in mediating fundamental cellular events such as transcription, recombination, replication and repair. The nuclear functions of HMGB1 are critical for survival as HMGB1^−/−^ mice are born alive but die within 24 h due to hypoglycaemia [8]. Moreover, cell lines lacking HMGB1 grow normally, but the activation of gene expression by different signal molecules is impaired [8].

More recently, it has been identified as extracellular signaling molecule able to induce different cell responses including cell motility, proliferation, differentiation, and cytokine/chemokine production [9,10]. 

HMGB1 can be released actively, by stimulated cells, or passively, by necrotic cells [11]. Particularly, “necrotic HMGB1” behaves as a damage-associated molecular pattern (DAMP) molecule, participating to initiation of reparative responses [12]. In contrast to necrosis, HMGB1 release seems to not occur during apoptosis [13], although Pisetsky and collaborators observed HMGB1 leaks out of apoptotic cells [14]. During the active release, HMGB1 undergoes different post-translational modifications, including hyper-acetylation, phosphorylation and ADP-ribosylation that facilitate HMGB1 dissociation from chromatin and, thus, its translocation to the cytoplasm where it is packaged into specialized secretory lisosomes [15,16,17].

This “non-classical” active release mechanism is Ca^2+^-dependent [18]. Moreover, some authors suggest that also the redox state of the released forms of HMGB1 is crucial for its extracellular activities [19].

The different effects exerted by extracellular HMGB1 on specific target cells have been related to the ability of this protein to interact with alternative cell surface receptors, such as the receptor for advanced glycation end products (RAGE) and the toll-like receptors (TLRs) 2 and 4 [20,21].

However, a number of observations indicate that HMGB1 can also act as a co-stimulating accessory protein without association with these receptors [11,22].

Wound repair is a process requiring local cellular and biochemical events, activated by several molecular mediators and interaction of numerous cell types. Growth factors and released cytokines during the wound play a crucial role in orchestrating the regenerative process which occurs during inflammation, proliferation and remodeling [23].

A number of observations indicate that all cell types involved in tissue-repair mechanism are sensitive to extracellular HMGB1 and express functional HMGB1 receptors. Indeed, this cytokine-like protein acts as chemoattractant and pro-motogenic/pro-mitogenic stimulus for fibroblasts, keratinocytes, endothelial cells, vascular smooth muscle cells and vessel-associated stem cells (mesangioblast) [24,25,26,27,28,29,30,31].

Moreover, HMGB1 can recruit neutrophils, monocytes and macrophages to flamed injured tissues, and it is able to induce the production and release of cytokines, chemokines and other important signal molecules by innate immune system cells [32].

A better understanding of HMGB1 role in tissue repair mechanisms can contribute to a further development of regenerative medicine.

## 2. High-mobility Group Box 1 (HMGB1) Structure

Structurally, HMGB1 consists of 215 residues organized in three main functional domains: the A and B boxes (positively charged) and the acidic tail (negatively charged). The A and B boxes, residues 1–79 and 89–163 respectively, are functionally DNA-binding domains [33,34].

The C-terminal acidic tail, residues 186–215, plays an important role in nuclear functions [35,36]. At the N-terminus of HMGB1 (residues 6–12) is an heparin-binding sequence that likely contributes to the heparin and heparin sulphate binding capacity of HMGB1. Recombinant analysis of the protein shows that B box contains the cytokine-like activity, inducing macrophage secretion of additional proinflammatory cytokines [37]. HMGB1 cytokine activity is antagonized by the recombinant A box, whereas in the recombinant B box the residues 89–109 represent the minimal peptide sequence with cytokine activity. Moreover, in the B box is located a short 10 amino acid peptide (residues 130–139), produced by proteolytic processing of extracellular HMGB1, active in erythroleukaemia cell differentiation [38]. Interestingly, this bioactive peptide is localized between the TLR4 and the RAGE binding sites identified in the B box of HMGB1 and doesn’t overlap with them. The RAGE binding motif is located between residues 150-183, downstream from peptide 130–139, whereas the TLR4 binding site is a 20-mer peptide stretch containing C106, upstream from peptide 130-139 [19,20].

Furthermore, in the primary structure are present three cysteine residues (C23, C45 and C106) crucial for the biological activities of extracellular HMGB1. To exert its cytokine-like functions, HMGB1 predominant form must contain a C106 thiol group and a disulfide bond between C23 and C45, whereas the inactive form of HMGB1 contains terminally oxidized cysteines [19,39]. Oxidation of HMGB1 could take place during pathological conditions, such as prolonged ischemia and liver transplantation, sterile injury, crush injury, and autoimmune-mediated tissue damage [19,40]. 

Interestingly, oxidative stress has been linked to aging, cancer and other diseases in humans [41]. Moreover, chemotactic activities of HMGB1 are associated with a different, yet to be characterized, pattern of cysteine redox modifications. It’s likely that “chemotactic HMGB1” corresponds to a fully reduced form, since recombinant HMGB1, supplemented with dithiothreitol (DTT), promotes cell migration, although it is unable to induce TNF production [39].

## 3. HMGB1 Receptors

The pleiotropic functions exerted by extracellular HMGB1 on specific target cells have been related to the ability of this protein to engage and activate different receptors, such as RAGE and TLRs 2, 4 and 9 [42]. RAGE is a member of the Ig superfamily of transmembrane protein and is expressed on several cell types, such as endothelial cells, vascular smooth muscle cells, monocytes and macrophages, dendritic cells, neurons and glial cells [43]. 

RAGE is a 35 kD transmembrane receptor of the immunoglobulin super family which was first characterized in 1992 by Neeper *et al.* [44]. Its name comes from its ability to bind advanced glycation end products (AGE), which include chiefly glycoproteins such as the glycans of which have been modified non-enzymatically through the Maillard reaction. 

RAGE engagement by HMGB1 leads to the activation of various signal transduction pathways, including small GTPases, mitogen activated protein kinases, stress activated protein kinases, and NK-kB, and culminates in cell responses ranging from cell motility to cell proliferation, including production and release of cytokines/chemokines. The K_d_ of the HMGB1/RAGE complex is approximately 10 nM [10]. 

Interestingly, RAGE blockade suppresses the HMGB1 effects, but only partially, strongly suggesting the existence of additional receptors for HMGB1. Indeed, the functional association of HMGB1 with some members of the TLRs family is widely documented. In this respect, both TLR2 and 4 are involved in HMGB1-induced pro-inflammatory activation of macrophages and neutrophils. Of note, HMGB1 alone is able to activate TLR4 (Yang and co-authors [19] observed an apparent K_d_ of the HMGB1/TLR4 of 1.5 μM), whereas for the activation of TLR2 and 9, HMGB1 has to form complexes with apoptotically released nucleosomes and ssDNA, respectively [42]. Since also TLRs blockade does not completely abolish HMGB1 effect and HMGB1 can exert some of its biological activities at sub-nanomolar concentrations (so far away from the Kd values for RAGE and TLR4), the possibility that other not yet identified HMGB1 receptors exist is actual. 

The identification of “new” receptors might be a crucial challenge in understanding HMGB1 biology. Moreover, a synergism between HMGB1 and LPS has been reported for TLR4 activation, as well as between HMGB1 and IL-1β in triggering inflammation through IL-1R engagement [22,45].

Recently, a new receptor mediating HMGB1 effects has been identified: Pedrazzi and colleagues [46] demonstrate that at concentrations of agonist per se ineffective, HMGB1 potentiates the activation of the ionotropic glutamate N-methyl-D-aspartate receptor (NMDAR), interacting with the ion channel through the sequence corresponding to the peptide located in the B box at the amino acids 130–139.

Thus, the biological activities of extracellular HMGB1 can be modulated at different levels including its release, the post-transductional modifications, the redox state, and the possibility to engage different receptors on the basis of its extracellular concentration, the presence of co-stimulatory molecules, and the K_d_ values of the signaling complexes.

## 4. Tissue Regeneration

During evolution, multicellular organisms have developed mechanisms to counteract life-threatening events such as infections and tissue injury, as well as to restore tissue homeostasis. These mechanisms are called “the inflammatory response”. To initiate an appropriate inflammatory response, organisms have developed ways to recognize potentially life-threatening events [47].

Trauma and tissue damage trigger an inflammatory response, which is required for post-injury regeneration and tissue repair. Inflammation following tissue damage is a dynamic process, which is driven by numerous inflammatory mediators [47]. Endogenous danger signals released from necrotic or stressed cells that trigger the inflammatory response after trauma have been termed alarmins or danger-associated molecular patterns (DAMPs) [48]. DAMPs share structural and functional similarities with exogenous, conserved microbial surface structures released from invading microorganisms, so-called pathogen-associated molecular patterns (PAMPs). However, this definition of DAMPs is not always used consistently, and sometimes endogenous alarmins and exogenous PAMPs are collectively classified as danger-associated molecular patterns (DAMPs). 

Well-known alarmins include but are not limited to heat shock proteins, hyaluronan, uric acid, galectins, thioredoxin, adenosine, HMGB1, interleukin-1α (IL-1α), and interleukin-33 (IL-33) [49]. As unique features, HMGB1, IL-1α, and IL-33 exert dual functions as intracellular transcription factors and as extracellular inflammatory mediators [50].

Several instances of HMGB1 as released by damaged tissues are well described. The damage can be caused not only by trauma, but also by the immune system itself [51].

Acute liver injury, such as that following ischemia and reperfusion of the organ, causes the rapid release of HMGB1, which triggers inflammation. Likewise, after the killing of hepatocytes by hepatitis virus B-specific cytotoxic T lymphocytes, HMGB1 directs the recruitment of neutrophils and of all others non-antigen specific inflammatory cells [52].

The role of HMGB1 as chemo attractant for inflammatory cells is thus out of the debate and the receptor involved in this phenomenon is the receptor for advanced glycation end product (RAGE). 

RAGE knock-out mice are viable and are less susceptible to sepsis. Some findings, such as the chemotactic responses in RAGE^−/−^ derived fibroblasts to HMGB1 [53], raised the possibilities that chemotactic and tissue repair activities of HMGB1 are not mediated only by RAGE.

It is also to note that among RAGE ligands, HMGB1 appears to play an important role in cancer. Many reports point out the significance of ligand/RAGE interactions in carcinogenesis, tumor progression and metastasis [54]. Some studies on the HMGB1/RAGE interactions revealed that this complex played a pivotal role in neurite outgrowth through activation of two small GTPases of the Rho family, Cdc42 and Rac1, thus suggesting that HMGB1/RAGE signaling might be associated with cancer metastasis [55]. HMGB1 was found to bind RAGE in a variety of cells such as neurons, endothelium, smooth muscle, monocytes, macrophages, T-cells and immature dendritic cells. Moreover, ligation of HMGB1 to RAGE triggered events such as endothelial cell activation [32]. 

HMGB1 fulfills the prediction that a signal from damaged tissue should promote their regeneration. In *in vitro* in absence of serum, HMGB1 acts as mitogen for several cell types, such as mesoangioblasts and endothelial precursor cells [26,56,57].

HMGB1 behaves as a trigger of tissue repair, recruiting stem cells, and promoting their proliferation [58]. Some studies on regenerative properties of HMGB1 have been performed on experimental models in skeletal and cardiac muscles. HMGB1 levels are increased in reparation of skeletal muscle and the intramuscular injection of HMGB1 increases the number of regenerating fibers [59]. Moreover, HMGB1 induces an increase of stem cells transmigration across the endothelial barrier. In addition, HMGB1 is able to induce myogenic differentiation via Cdc42-Rac1-MAPKs. HMGB1 stimulates also the migration of rat smooth cells, inducing rapid and transient changes in cell shape and actin reorganizations [60].

HMGB1 also promotes heart regeneration after infarction. Injection of HMGB1 into mouse hearts after ischaemic damage resulted in the formation of new myocytes. Indeed, HMGB1 induced c-kit+ cardiac stem-cell proliferation and differentiation. Interestingly, differentiation towards the myocardial lineage was not observed in non-infarcted HMGB1-treated hearts, suggesting that signals released from damaged tissue are required for differentiation [61].

HMGB1 is also necessary for the transfilter migration of glioma cells and that migration is inhibited by antibodies against the RAGE-binding domain of HMGB1. By studying nerve crush injuries, Rauvala and coworkers determined that HMGB1 messenger RNA is present in cell bodies before injury but is only translated into protein after injury. Thus, it is possible to postulate that HMGB1 is a danger signal that is locally translated in injured axons to enhance regeneration of the nerve processes [62].

With regards to angiogenesis, HMGB1 is able to induce the formation of new blood vessels through various pathways, including upregulation of pro-angiogenetic factors, promoting the homing of endothelial progenitor cells and sprouting [63].

Given its potent extracellular functions, it is likely that HMGB1 is counteracted by inhibiting molecules. Endothelial thrombomodulin binds HMGB1 through its lectin-binding domain and thereby dampens inflammation. Thrombomodulin inhibited HMGB1-induced NF-κB translocation *in vitro* and decreased ultraviolet-induced skin inflammation *in vivo* [64].

Cytokine-induced cell proliferation and migration are also fundamental elements of skin repair. Growth factors are thought to be main intercellular signaling that orchestrates the complex sequence of wound healing-related cell activities [47]. Due to the observed pro-inflammatory role of HMGB1, and its ability to induce cell proliferation and motility, HMGB1 has been proven to possess this kind of property on keratinocytes and fibroblasts [24,25].

The physiological meaning of this property in cutaneous wound healing remains to be ascertained. Straino and coworkers [50] have shown that HMGB1 levels are reduced in the skin of both diabetic human and mice, having impaired tissue repair and report different mechanisms by which HMGB1 may promote wound healing. These findings fit a recent view assuming that many elements, such as HMGB1, could be combined to recreate a receptive environment (or “soil”), which in combination with the appropriate stem cells (or “seed”) would provide a force for tissue repair and regeneration [65].

So, it is possible to postulate that HMGB1 release may occur *in vivo* from skin cells, such as fibroblasts and keratinocytes, as the result of cell activation, enhanced membrane permeability, or membrane ruptures. After its release in the wounded area, HMGB1 could operate the recruitment of different cell types, and influence keratinocyte and fibroblast proliferation and migration [24,25].

HMGB1 could trigger and strengthen the healing cascade and this could be relevant to long-term effects on wound healing and tissue repair. These kind of processes are of great interest for the treatment of tissue lesions occurring in certain diseases, e.g., diabetes, in which cells are shown to be less responsive to growth factors [66].

Of particular interest is the HMGB1 sensitivity to oxidation [67]. Oxidative stress is an early player in acute inflammatory response; it elicits the formation of reversible covalent disulfide bonds between thiols [68]. HMGB1 contains three cysteine residues at positions 23, 45 and 106. Mild oxidation status induces the formation of an intramolecular disulfide bond between cysteines in position 23 and 45. These residues are also implicated in the binding of HMGB1 to Toll-like receptor 4 [69]. 

An oxidized environment could modulate HMGB1 functions. Some authors have suggested that oxidation could quench HMGB1 inflammatory effects. In this scenario, a reduced environment could sustain and prolong HMGB1 bioactivity, but further studies are needed to verify these hypotheses.

## 5. Conclusions

The dual-function of HMGB1 represents a crucial mediator in the initiation and perpetuation of the inflammatory response following loss of cellular integrity. 

Basic research as well as clinical studies is strongly needed to further unravel the complexity of the host response after trauma and tissue damage. 

Novel preclinical models of trauma may help characterize the role of DAMPs and investigate mechanisms/kinetics of release after tissue injury in single-organ injury and multi-system trauma. 

In addition, little is known about mutual interactions of DAMPs prior and after passive or active release and direct crosstalk with other mediators of inflammation and signaling systems. 

As regards potential therapeutic strategies, alongside agents that neutralize or block DAMPs, the development of compounds that cause intracellular DAMP retention and limit DAMP release upon tissue damage might represent a promising approach. 
